# A longevity-associated variant of the human *BPIFB4* gene prevents diastolic dysfunction in progeria mice

**DOI:** 10.1038/s41392-025-02416-3

**Published:** 2025-09-29

**Authors:** Yan Qiu, Monica Cattaneo, Anna Maciag, Annibale A. Puca, Paolo Madeddu

**Affiliations:** 1 Bristol Heart Institute, University of Bristol, Bristol, UK; 2 Cardiovascular Department, IRCCS MultiMedica, Milan, Italy; 3 Department of Medicine, Surgery and Dentistry, University of Salerno, Salerno, Italy

**Keywords:** Experimental models of disease, Cardiology, Cardiovascular diseases, Translational research

**Dear Editor**,

Hutchinson-Gilford Progeria Syndrome (HGPS) is caused by a mutation in the Lamin A/C gene (*LMNA*), resulting in the synthesis and accumulation of an abnormal protein, progerin, which disrupts the structural integrity and function of the nucleus and nucleolus. Affected individuals exhibit a senescent phenotype and die prematurely due to cardiovascular complications.^1^ The exceptional case of Sammy Basso, who lived until the age of 28, has brought renewed public attention to this rare disease.

Lonafarnib, a farnesyltransferase inhibitor that prevents the synthesis and accumulation of progerin, is the sole FDA-approved treatment for HGPS. In October 2024, a phase 2a clinical trial of Lonafarnib in combination with Progerinin, an improved progerin-Lamin A binding inhibitor, was started in a small cohort of children affected by HGPS. New cardiovascular treatments that can extend life until a definitive solution from DNA base editing are urgently needed.

We previously demonstrated that supplementing with the longevity variant (LAV) of the BPI fold containing family B, member 4 (BPIFB4), found in healthy supercentenarians, can delay and even reverse the ageing clock in animal models of cardiovascular disease.^2^ Supercentenarians’ cells exhibit minimal alterations in nuclear envelope components and possess a distinctive resilience to prelamin A accumulation.^3^ The other way round, progerin inhibits the nuclear translocation of Transcription Factor EB (TFEB), a transcriptional activator of *BPIFB4* and a regulator of autophagy and lysosomal biogenesis.^4^ Based on this background, we elaborated the new hypothesis that *BPIFB4* is downregulated in HGPS cells and that the transfer of the *BPIFB4* gene protects from *LMNA* mutation-induced aging.

First, we determined whether *LAV-BPIFB4* horizontal transfer could improve early cardiomyopathy in hemizygous transgenic C57BL/6-Tg(LMNA*G608G)HClns/J mice, which mimic the early cardiovascular traits of human HGPS. Second, *LAV-BPIFB4* was tested on HGPS patient fibroblasts in vitro. Results are illustrated in Fig. 1 and the online Repository Supplement (https://zenodo.org/records/16792599).

**Fig. 1:**
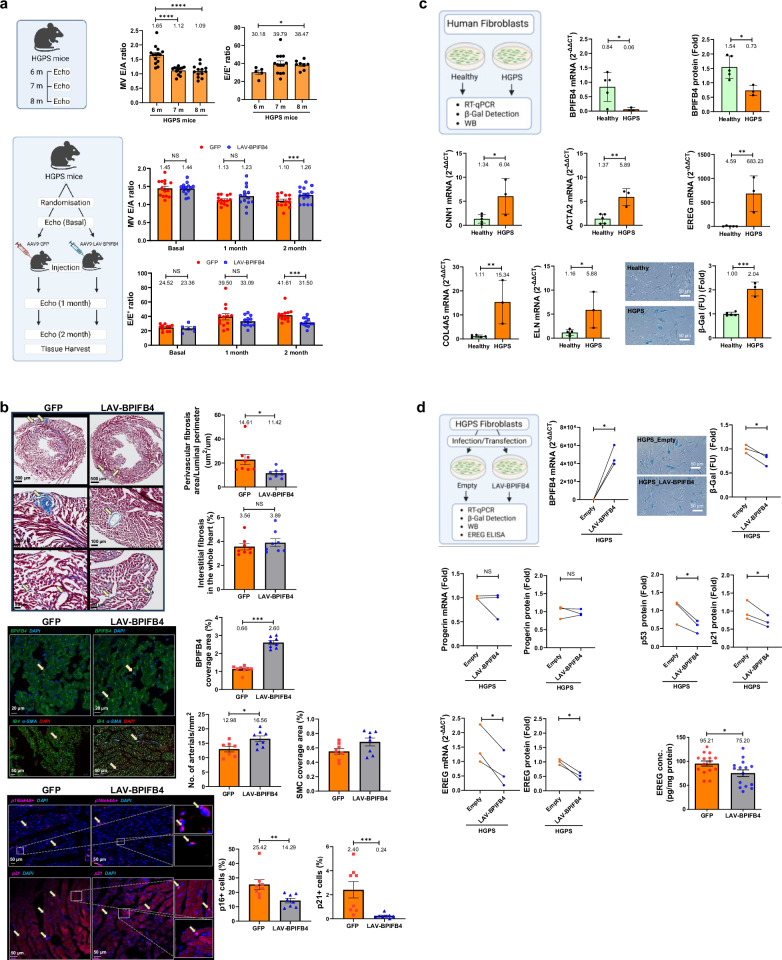
**a** Progeria mice exhibit early cardiac diastolic dysfunction, which is attenuated by *AAV9-LAV-BPIFB4* gene transfer. (Top) Heart function was assessed using echocardiography in 6–8-month-old progeria mice. Diastolic function was determined with the E/A (mitral valve peak E and peak A waves ratio) and E/E’ (mitral valve peak E wave and mitral annular peak E’ wave ratio). Then, we studied the same parameters in progeria mice that received *AAV9-GFP* or *AAV9-LAV-BPIFB4* (Bottom). GFP: *AAV9-GFP*-treated mice; LAV-BPIFB4: *AAV9-LAV-BPIFB4*-treated mice. Two months post the *AAV9-GFP* or *AAV9-LAV-BPIFB4* injections, the E/A and E/E’ indexes were determined (*n* = 13–14 for E/A at each time point; *n* = 6–11 for E/E’ at baseline and 12–14 for 1- and 2-month post-injection). **b** Treatment with *AAV9-LAV-BPIFB4* reduces cardiac perivascular fibrosis and improves cardiac microvasculature in progeria mice. (Top) Fibrosis was determined by collagen staining in the whole left ventricle of *AAV9-GFP-* and *AAV9-LAV-BPIFB4*-treated mice two months after virus injection (*n* = 8 for *AAV9-GFP- and AAV9-LAV-BPIFB4*-treated progeria mice). Bar graphs summarize the quantitative analysis of the perivascular fibrosis index of subepicardial arteries, expressed as the perivascular fibrosis area standardized against the luminal perimeter in male and female combined groups. The treatment did not affect interstitial fibrosis. (Middle) The BPIFB4 expression was expressed as the percentage of BPIFB4 coverage area in total tissue area in each image. The number of microvessels (IB4, green) and arterials (a-SMA, blue), as well as the coverage area of microvessels and a-SMA+ cells, were normalized against the total tissue area in each image (nuclei stained with DAPI, red). In addition, (Bottom) treatment with *AAV9-LAV-BPIFB4* reduced senescence in the heart. The percentage of senescent cells in the whole left ventricle in *AAV9-GFP-* and *AAV9-LAV-BPIFB4-*treated mice was determined, with the data reported as the percentage of p16+ cells (pink) or p21+ cells (pink) relative to the total cell number (nuclei stained with DAPI, blue). GFP: *AAV9-GFP*-treated mice; LAV-BPIFB4: *AAV9-LAV-BPIFB4*-treated mice. **c** HGPS patient fibroblasts exhibit downregulation of endogenous BPIFB4 expression, activation of typical myofibroblast genes and increased senescence. (*n* = 5 for healthy controls and 3 for HGPS patients). (Top) Endogenous BPIFB4 mRNA and protein levels were assayed by RT-qPCR and Western blot with corresponding densitometric quantification. (Middle and Bottom, left) Genes associated with myofibroblast activation were assayed by RT-qPCR. In addition, (Bottom, right) senescent fibroblasts were identified by histochemical staining and quantitative β–Galactosidase assay. Healthy: fibroblasts from healthy controls; HGPS: fibroblasts from HGPS patients. **d** Ectopic expression of LAV-BPIFB4 normalizes HGPS patient fibroblasts (*n* = 3). (Top, left) BPIFB4 expression was increased by vector-mediated transfection of HGPS fibroblasts. (Top, right) Staining and measurement of β-Galactosidase levels were carried out in HGPS fibroblasts transfected with *LAV-BPIFB4*. (Middle) HGPS fibroblasts infected with *LAV-BPIFB4* lentivirus were analyzed for progerin mRNA and protein by semi-quantitative PCR and Western blot, respectively. Senescence in *LAV-BPIFB4*-infected HGPS fibroblasts was evaluated by Western blot analysis of p53 and p21, with relative quantification. (Bottom, left and center) EREG transcript and protein released into the supernatant from *LAV-BPIFB4*-infected HGPS fibroblasts were measured by RT-qPCR and ELISA assay, respectively. Empty: HGPS fibroblasts transfected/infected with empty vectors; LAV-BPIFB4: HGPS fibroblasts transfected/infected with vectors containing *LAV-BPIFB4*. Furthermore, (Bottom, right) EREG protein expression was assessed in cardiac tissues of *AAV9-GFP-* and *AAV9-LAV-BPIFB4*-treated mice two months post-virus injections**;** *AAV9-LAV-BPIFB4* reduced EREG expression in the progeria mouse heart. β–Gal: β–Galactosidase, EREG: epiregulin. Data are represented as mean ± SEM. The descriptive scientific graphics were created using BioRender and the corresponding licences are detailed in the online Repository Supplement

Like HGPS children, 26-week-old progeria mice have growth retardation as compared with age-matched normal C57Bl/6 mice (Jackson Laboratories dataset, online Repository Supplementary Fig. 1a, b). Three-dimensional echocardiography showed that untreated progeria mice spontaneously acquire left ventricular (LV) diastolic dysfunction at 7 or 8 months of age, as demonstrated by a decreased E/A ratio and higher E/E’ ratio (Fig. 1a). In contrast, systolic function parameters remained unchanged (online Repository Supplementary Fig. 2a–e). Prakash et al. found diastolic dysfunction as the first echocardiographic abnormality in children with HGPS. They hypothesized that myocardial fibrosis may be the structural cause of altered LV relaxation.^1^

In a randomized study, male and female progeria mice received a single intraperitoneal injection of *LAV-BPIFB4* or *GFP* via an adeno-associated subtype-9 viral vector (AAV9, 1 × 10^12^ GC/ml in 100 μl PBS). *AAV9-LAV-BPIFB4* enhanced cardiac BPIFB4 protein expression 2.5-fold and boosted female mice’s body weight (online Repository Supplementary Fig. 1c, d). *AAV9-LAV-BPIFB4* significantly reduced age-related changes in LV diastolic function compared to *AAV9-GFP*-treated controls (Fig. 1a), but did not affect systolic function or LV mass (online Repository Supplementary Fig. 3a–e).

In progeria mice, histological analyses demonstrated that *AAV9-LAV-BPIFB4* decreased perivascular fibrosis, but not interstitial fibrosis. Moreover, as shown using fluorescent immunohistochemistry, gene therapy increased the expression of BPIFB4, as well as the number of coronary arterioles along with vascular smooth muscle cell coverage, while reducing cardiac p16- or p21-positive senescent cells (Fig. 1b). The anti-fibrotic, pro-survival, and vascular actions of *AAV9-LAV-BPIFB4* may account for improving diastolic dysfunction.

Aortas were harvested and examined after HGPS mice were culled after 2-month follow up and there was no evidence of atherosclerotic lesions in the aortas of 8-month-old progeria animals. This, along with the liver’s modest collagen deposition, suggests that the model is an early form of HGPS. On the other end, senescent cells, identified by p16 or p21 markers, were abundant in the livers of progeria mice. *AAV9-LAV-BPIFB4* decreased p21-positive senescent cells but not p16-positive ones (online Repository Supplementary Fig. 4).

We studied fibroblast cell lines from three HGPS patients and five unaffected parents to investigate the effects of *LAV-BPIFB4* on fibrosis and senescence. All cell lines expressed Lamin A/C, but only HGPS fibroblasts expressed progerin mRNA and protein (online Repository Supplementary Fig. 5). HGPS fibroblasts exhibited lower *BPIFB4* mRNA and protein levels along with marked increases in calponin-1 (*CNN1*), smooth muscle α-actin (*ACTA2*), and epiregulin (*EREG*) as well as pro-fibrotic and senescent markers, such as Col4A5 and Elastin, and β-galactosidase (Fig. 1c). Lentivirus infection or plasmid transfection of *LAV-BPIFB4* reduced senescence, p53 and p21 levels, and *EREG* mRNA and protein secretion into the medium without affecting progerin accumulation (Fig. 1d). The cardiac tissue of progeria animals treated with *AAV9-LAV-BPIFB4* exhibited lower EREG levels, consistent with the in vitro results (Fig. 1d). These data indicate that LAV-BPIFB4 reduces cellular senescence via the p53–p21 and p16 axes and modulates early fibrotic signaling via the EREG ligand of the Epidermal Growth Factor Receptor (EGFR) pathway. The treatment did not directly affect terminal myofibroblast differentiation or extracellular matrix deposition. Altogether, results indicate that *LAV-BPIFB4* decreases progerin toxicity rather than suppressing this abnormal protein.

Our study is the first to demonstrate that a supercentenarian gene mutation protects the HGPS heart against fibrosis and promotes cell survival, thereby preserving diastolic function. Mechanistic investigations on HGPS fibroblasts showed lower endogenous BPIFB4 expression, probably due to progerin-induced transcriptional suppression, and an enhanced aged and fibrotic phenotype, which was rescued by exogenous *LAV-BPIFB4*.

*LAV-BPIFB4* transduction may affect nucleolar activities, including ribosome biogenesis and ribonucleoprotein assembly, downstream of the LMNA mutation, resulting in therapeutic actions.^5^ The creation of an interactive complex with a nucleolar RNA-binding protein that affects ribosome assembly, biogenesis, DNA transcription, and telomere preservation and repair represent some of LAV-BPIFB4’s known molecular functions.^2^ LAV-BPIFB4 may also protect cardiomyocytes through paracrine signaling systems, as shown by its favorable chronotropic and inotropic effects on human iPSC-derived cardiomyocytes.^2^

Other gene variations contribute to healthy aging than *LAV-BPIFB4*. The genetics of supercentenarians may therefore disclose new global treatments for premature or accelerated cardiac senescence.

## Supplementary information

Supplementary Materials_4th revision_YQ

## Data Availability

The authors declare that all data supporting the findings of this study are available within the article, its Supplementary Materials file and the online Repository Supplement (https://zenodo.org/records/16792599).
