# Supplementary material for: A longevity-associated variant of the human BPIFB4 gene prevents diastolic dysfunction in progeria mice
Source: Signal Transduct Target Ther. 2025 Sep 29;10:314. doi: 10.1038/s41392-025-02416-3 (PMC12480688; doi:10.1038/s41392-025-02416-3)
Supplement: Supplementary file 1 — Supplementary Materials_4th revision_YQ [file 41392_2025_2416_MOESM1_ESM.docx]

Supplementary Materials for

**A longevity-associated variant of the human *BPIFB4* gene prevents diastolic dysfunction in progeria mice**

Authors: Yan Qiu^1#^, Monica Cattaneo^2#^, Anna Maciag^2^, Annibale A Puca^2, 3*^, Paolo Madeddu^1*^

# Authors who equally contributed to the study

* Both are senior authors

Correspondence to: [y.qiu@bristol.ac.uk](mailto:y.qiu@bristol.ac.uk) and annibale.puca@multimedica.it

**This PDF file includes:**

Materials and Methods

Materials and Methods

Animal model

All animal procedures conformed to the guidelines from Directive 2010/63/EU of the European Parliament on protecting animals used for scientific purposes and were approved by the University of Bristol and the British Home Office (PPL: PP1377882).

Mice purchased from a certified vendor (Charles River, UK) were housed in custom-made facilities at the University of Bristol’s Animal Services Unit (ASU). They were fed a standard diet and had free access to water ad libitum. One week of acclimatization to the facilities was allowed before starting the experimental protocol.

Six-month-old male and female hemizygous transgenic C57BL/6-Tg (LMNA G608G) HClns/J/J mice, recapitulating the early cardiovascular features of HGPS, were randomized to receive a single intraperitoneal injection of *AAV9-LAV-BPIFB4* or *AAV9-GFP* in PBS *(*100 μl of 1×10^12^ GC/ml).[1] Virus production and purification were carried out as described previously.[1]

Echocardiographic measurements

Heart function was monitored at baseline, 1 month, and 2 months after virus injection using a 3D Vevo 3100 echocardiograph (Visual Sonics, Toronto, Canada) as described previously.[2] All measurements were performed with mice under 1-3% isoflurane anesthesia and a heart rate of 390 - 410 beats per minute for diastolic function, and 420 – 480 beats per minute for systolic function monitoring. In brief, diastolic function was assessed using pulsed wave Doppler and tissue Doppler to calculate the E/A and E/E’ ratios. M-mode images were used to calculate the systolic function indexes: ejection fraction (EF), fractional shortening (FS), and cardiac output (CO). In addition, left ventricular (LV) mass was calculated based on the anatomical measurement with echocardiography and was presented without normalization for tibia length (TL) because this model frequently exhibits skeletal abnormalities such as bone shortening.

Tissue collection

At the end of the last echocardiography session, isoflurane anesthetized mice were perfused through the apex of the left ventricle with 1 ml ice-cold potassium chloride (30 mM), to stop the heart in diastole, followed by 1% EDTA in normal phosphate-buffered saline (PBS) with a pump pressure at 100-110 mmHg. When the heart expanded, the right atrium was snipped, and blood was collected in 1.5 ml Eppendorf tubes coated with heparin (764 USP units/ml). The top section of the ventricle was flash-frozen, and the lower section was drop-fixed in 4% PFA. Pieces of liver and aorta were also flash-frozen and drop-fixed in 4% PFA. All PFA samples were kept at 4^o^C for 18-24 hours, and then the PFA was replaced with PBS. The PFA-fixed tissues were cryoprotected using 30% sucrose for 24 hours before being embedded in OCT. Histochemical and immunohistochemical studies were performed on 5-μm thick sections.

Determination of BPIFB4 expression in heart tissue

As described previously,[3] antigen retrieval was performed using citrate buffer (pH = 6; 1x; Sigma‒Aldrich) for 15 minutes at 98°. A rabbit anti-BPIFB4 antibody (1:100, GeneTex, #GTX51455) and Alexa Fluor 488-conjugated anti-rabbit antibody (1:500) were used for BPIFB4 visualization. Nuclei were labeled with DAPI (1:1000). Six random images from each heart section were taken via a Leica TCS-SP8 confocal laser scanning microscope attached to a Leica DM I8 inverted epifluorescence microscope and processed via LASX software. The BPIFB4 coverage area was analyzed using ImageJ software.

Determination of microvascular density in mouse heart tissue

As described previously,[4] heart sections were stained with antibodies against α-SMA actin (1:400, Sigma, #C61198) and isolectin-B4 (1:200, Life Technologies, #121414) to identify vascular smooth muscle cells and endothelial cells. Immunofluorescent profiles of capillaries and arterioles were captured with a slide scanner (Olympus SLIDEVIEW VS200) with a 20x objective lens to calculate vascular density, expressed as the number of capillaries and arterioles per mm^2^.

Determination of fibrosis in heart and liver tissues

Heart and liver sections were stained for collagen using the Azan Mallory method (Heidenhain’s adaptation of Mallory’s trichrome stain). Images were captured with a slide scanner (Olympus SLIDEVIEW VS200) with a 20x objective lens. The fibrosis content was assessed in the perivascular area of the heart and quantified as a ratio between the collagen area and vessel luminal perimeter. At the same time, interstitial fibrosis of the heart was measured in pixels and expressed as a percentage of tissue area.

Determination of cell senescence in mouse heart, liver and aorta tissues

As described previously,[4] senescent cells were identified in the heart, liver and aorta sections using anti-mouse p16ink4A (1:50, Santa-Cruz, #sc-1661) or anti-rabbit p21 (1:100, Abcam, #ab188224) and expressed as the percentage of positive nuclei. Alexa Fluor 564-conjugated anti-mouse IgM (1:500, Life Technologies, UK) and Alexa Fluor 488-conjugated anti-rabbit IgG (1:500, Life Technologies, UK) were used as secondary antibodies. Images were captured using a Zeiss Observer or Olympus SLIDEVIEW VS200 slide scanner with a 20x objective lens.

Cell culture and *LAV-BPIFB4* treatment

Primary human dermal fibroblast cell lines were obtained from the Progeria Research Foundation. Three cell lines were from patients with the classic mutation in LMNA Exon 11, heterozygous c.1824C > T (p.Gly608Gly), and five from unaffected healthy parents. The list of the cell lines, together with the source and the age at donation, are listed in online Repository Supplement (<https://zenodo.org/records/16792599>) Table 1. For all experiments, cells were passage-matched between P12-14. Cells were grown in high glucose DMEM containing 15% fetal bovine serum (FBS), 1% GlutaMAX (ThermoFisher, #35050-061) and 1% Penicillin-Streptomycin (ThermoFisher, #15140-122) at 37°C in 5% CO_2_.

Lentiviral particles (Empty vector or *LAV-BPIFB4*) were generated as previously described.[4] Lentiviral particles were concentrated by ultracentrifugation (40,000 rpm for 2 hours at 4 °C) and stored at − 80 °C until use. Lentivirus titration was performed by transducing HEK293T cells with concentrated particles in the presence of 4 µg/ml polybrene and measuring GFP expression after 3 days by flow cytometry. Cells were plated into a 12-well plate at a density of 500,000 cells/well and infected with empty lentiviral vector or particles encoding *LAV-BPIFB4* at 5 multiplicities of infection (MOI). After 72 hours, cells were selected with 1 µg/ml puromycin for 48 hours.

Cells were transfected with the indicated plasmids (pcmv6-myc carrying *LAV-BPIFB4* or empty vector) using Lipofectamine 3000 (Invitrogen) according to the manufacturer’s instruction and harvested 48 hours after transfection.

RNA extraction, reverse transcription, polymerase chain reaction (qPCR) and quantitative Real-Time PCR

Total RNA was extracted with RNeasy (Qiagen, USA), following the manufacturer's protocol. Total RNA concentration and quality were determined using a Nanodrop spectrophotometer (Nanodrop 1000, Thermo Fisher Scientific). Potential genomic DNA contamination was removed by DNase I treatment (Thermo Fisher Scientific). Subsequently, cDNA was synthesized with Superscript VILO cDNA synthesis kit (Thermo Fisher Scientific), following the manufacturer’s protocol. PCR amplification was performed using GoTaq DNA Polymerase (Promega, Milan, Italy). The PCR conditions for LMNA and progerin were 95°C for 2 minutes, followed by 35 cycles at 95°C for 30 seconds, 60°C for 40 seconds, and 72°C for 60 seconds.

Quantitative Real-Time PCR was conducted with QuantStudioTM 6 Flex Real-Time PCR System (Applied Biosystems) with SYBR Green PCR Master Mix (Applied Biosystems, Life Technology). Expression levels were normalized to 18S. Primer sequences are listed in online Repository Supplement Table 2. Data was expressed as 2^-(ΔΔCt)^.

Western blotting

Cells were lysed in RIPA buffer containing protease and phosphatase inhibitor cocktails (Sigma-Aldrich). Protein concentration was determined using the Bradford assay (Sigma-Aldrich). Total protein was separated by electrophoresis using 4–12% NuPAGE Bis-Tris protein gels (Thermo Fisher Scientific), transferred onto a polyvinylidene difluoride (PVDF) membrane (GE Healthcare, Buckinghamshire, UK), and probed with indicated antibodies. Blots were visualized with ECL for detecting the chemiluminescent signal using UVITEC Alliance Q9 (Cambridge, UK). Densitometric quantification was normalized to beta actin signal using Q9 Alliance software (Uvitec). Antibody information is listed in online Repository Supplement Table 3.

EREG measurements with enzyme-linked immunosorbent assay (ELISA)

Cells were cultured in six-well plates and serum-starved for 24 hours. The EREG concentration was measured in starved supernatant by ELISA kits (Abcam, Cambridge, UK), according to the manufacturer’s instructions. The amount of the extracellular EREG was normalized to the total protein concentration. To quantify the expression levels of EREG in the mouse heart, the protein was extracted from frozen heart tissues, quantified, and EREG levels were assayed with the ELISA kit (Insight Biotechnology, UK. #ELM-Epiregulin-1), according to the manufacturer’s instructions. EREG concentration was normalized to the total protein concentration.

Determination of fibroblast senescence

Senescence-associated biomarker beta-galactosidase (β-Gal) was detected using senescence cells histochemical staining kit (Sigma) and β-Gal Detection Kit (Abcam, Cambridge, UK), according to the manufacturer’s instructions. The fluorescence intensity of the fluorogenic fluorescein digalactoside product was measured using a microplate reader at Ex490/Em525 nm (BioTeck, Synergy 2, Winooski, USA) and normalized to the total protein concentration.

Statistical Analysis

All statistical analyses were performed with Prism version 10 (GraphPad Software, San Diego, CA, USA). Data was first assessed for normal distribution using the Shapiro-Wilk test. Statistical significance was determined using an unpaired/paired t-test for normally distributed data with two groups. Non-normally distributed data was assessed using a Mann-Whitney test. For data with more than two groups, normally distributed data was assessed using ANOVA followed by Šídák's multiple comparisons test to compare the means of selected groups. Non-normally distributed data was assessed using the Kruskal-Wallis and Dunn’s multiple comparison tests. Echocardiography data collected at baseline and during follow-up was evaluated using a mixed-effects model, two-way ANOVA, which examined the influence of two categorical independent variables (treatment and sex) on one continuous dependent variable and then to determine if they were interacting. Where sex caused no significant difference, data from male and female mice were pooled for further analysis.

**References**

1. Villa, F., et al., *Genetic Analysis Reveals a Longevity-Associated Protein Modulating Endothelial Function and Angiogenesis.* Circ Res, 2015. **117**(4): p. 333-45.

2. Qiu, Y., et al., *Endothelial glycocalyx is damaged in diabetic cardiomyopathy: angiopoietin 1 restores glycocalyx and improves diastolic function in mice.* Diabetologia, 2022. **65**(5): p. 879-894.

3. Alvino, V.V., et al., *Healthy longevity-associated protein improves cardiac function in murine models of cardiomyopathy with preserved ejection fraction.* Cardiovasc Diabetol, 2024. **23**(1): p. 397.

4. Cattaneo, M., et al., *The longevity-associated BPIFB4 gene supports cardiac function and vascularization in ageing cardiomyopathy.* Cardiovasc Res, 2023. **119**(7): p. 1583-1595.
